# Long Noncoding RNA ZBED5-AS1 Facilitates Tumor Progression and Metastasis in Lung Adenocarcinoma via ZNF146/ATR/Chk1 Axis

**DOI:** 10.3390/ijms241813925

**Published:** 2023-09-10

**Authors:** Feng Jiang, Xiaolu Huang, Liqun Ling, Shiyi Tang, Huixin Zhou, Xueding Cai, Yumin Wang

**Affiliations:** 1 Department of Laboratory Medicine, The First Affiliated Hospital of Wenzhou Medical University, Wenzhou 325015, China; jiangfeng5945@126.com (F.J.);; 2 Key Laboratory of Clinical Laboratory Diagnosis and Translational Research of Zhejiang Province, Wenzhou 325015, China; 3 Department of Respiration, The First Affiliated Hospital of Wenzhou Medical University, Wenzhou 325015, China

**Keywords:** lung adenocarcinoma, long noncoding ZBED5-AS1, exosomes, proliferation, metastasis

## Abstract

Long noncoding RNAs (lncRNAs) have been implicated in tumorigenesis, including lung adenocarcinoma (LUAD). However, the functional and regulatory mechanisms of lncRNAs in LUAD remain poorly understood. In this study, we investigated the role of lncRNA ZBED5-AS1 in LUAD. We found that ZBED5-AS1 was upregulated in LUAD specimens and overexpressed in LUAD cell lines. ZBED5-AS1 promoted LUAD cell proliferation, migration, and invasion in vitro and promoted LUAD cell growth in vivo. ZBED5-AS1 promoted ZNF146 expression, activating the ATR/Chk1 pathway and leading to LUAD progression. We observed that exosomes from LUAD cells have a higher expression of ZBED5-AS1 compared with exosomes from the normal cell line BEAS-2B. Coculture experiments with exosomes showed that ZBED5-AS1 expression was downregulated after coculture with Si-ZBED5-AS1 exosomes, and coculture with exosomes with low ZBED5-AS1 expression inhibited proliferation and invasion of LUAD cells. Our results indicate that ZBED5-AS1 functions as an oncogenic factor in LUAD cells by targeting the ZNF146/ATR/Chk1 axis.

## 1. Introduction

Lung cancer is a common and lethal malignancy that accounts for most cancer-related deaths globally [1], with lung adenocarcinoma (LUAD) accounting for approximately 40% of lung cancers [2]. Due to nonspecific symptoms, limited diagnostic options, and lack of expression of reliable tumor markers during the early stages, most patients are diagnosed at a late stage [3]. Despite advances in lung cancer biology, the development of innovative diagnostic techniques, and improved therapeutic strategies, LUAD prognosis remains disappointingly poor, highlighting the need to identify novel diagnostic and therapeutic strategies [4].

Long noncoding RNAs (lncRNAs; >200 nucleotides) are involved in several pathological and physiological processes [5]. Accumulating evidence suggests that lncRNAs play key roles in cancer biology, and dysregulated lncRNA expression is associated with multiple cellular processes in cancers, such as cell proliferation, migration, and apoptosis [6]. LncRNAs are also considered prognostic or diagnostic markers in LUAD. For instance, upregulation of LncRNA-AC02278.4 has been demonstrated to regulate cell proliferation and migration in LUAD and worsen lung cancer prognosis [7].

ZBED5-AS1 is a lncRNA located on chromosome 11p15.4. A recent research study has hinted at a potential link between ZBED5-AS1 and the development of ovarian cancer, as predicted by bioinformatics analysis [8]. Our research group has previously reported that ZBED5-AS1 is differentially expressed in exosomes of pleural effusion of LUAD patients and healthy controls. Additionally, we found that ZBED5-AS1 plays an active role in promoting the proliferation, migration, invasion, and colony formation of LUAD cells [9]. However, the molecular mechanisms underlying LUAD development and its association with lncRNA ZBED5-AS1 remain unclear. Zinc finger protein 146 (ZNF146) is a 33 kDa protein consisting of 10 zinc finger motifs. ZNF146 acts as an oncogene that promotes gastric cancer [10]. It is also overexpressed in most pancreatic cancers and in >80% of colorectal cancers. ZNF146 expression occurs early in the progression of colorectal cancer [10,11] and is a target of the oncogene c-Myc [12]. ATR-Chk1 is one of the key pathways activated in lung cancer cells in response to DNA damage [13]. The ATR/Chk1 signaling pathway is often upregulated in cancer and promotes tumor growth; consistently, ATR and Chk1 inhibitors kill tumor cells [14]. Chk1 inhibition using genotoxic drugs can suppress DNA damage and replication checkpoint responses, thereby enhancing tumor cell killing [15]. Furthermore, inhibition of the ATR/Chk1 pathway can inhibit apoptosis, invasion, and migration [16]. However, as it is a novel axis, its mechanisms require further examination.

Exosomes, which are extracellular vesicles 30–150 nm in diameter, promote cell–cell communication by transferring parent cell-derived cargo comprising biomolecules such as proteins and RNAs [17]. Several studies have implicated the role of exosome-mediated transfer of particular lncRNAs in cancer progression [18]. Therefore, exosomal lncRNAs have attracted considerable interest in cancer research.

The aim of the present study was to elucidate the molecular mechanisms above and the role of lncRNA ZBED5-AS1 in LUAD development. Additionally, we investigated the potential functional roles of exosomal lncRNA ZBED5-AS1 in LUAD. Our study demonstrates that upregulation of ZBED5-AS1 enhances the proliferation, migration, and invasion of LUAD cells through the EMT pathway by activating ZNF146/ATR/CHK1 axis. Additionally, we found that exosomes containing Si-ZBED5-AS1 can reduce the invasive, migratory, and proliferative abilities of LUAD cells.

## 2. Results

### 2.1. ZBED5-AS1 Is Highly Expressed in LUAD

**Figure 1:**
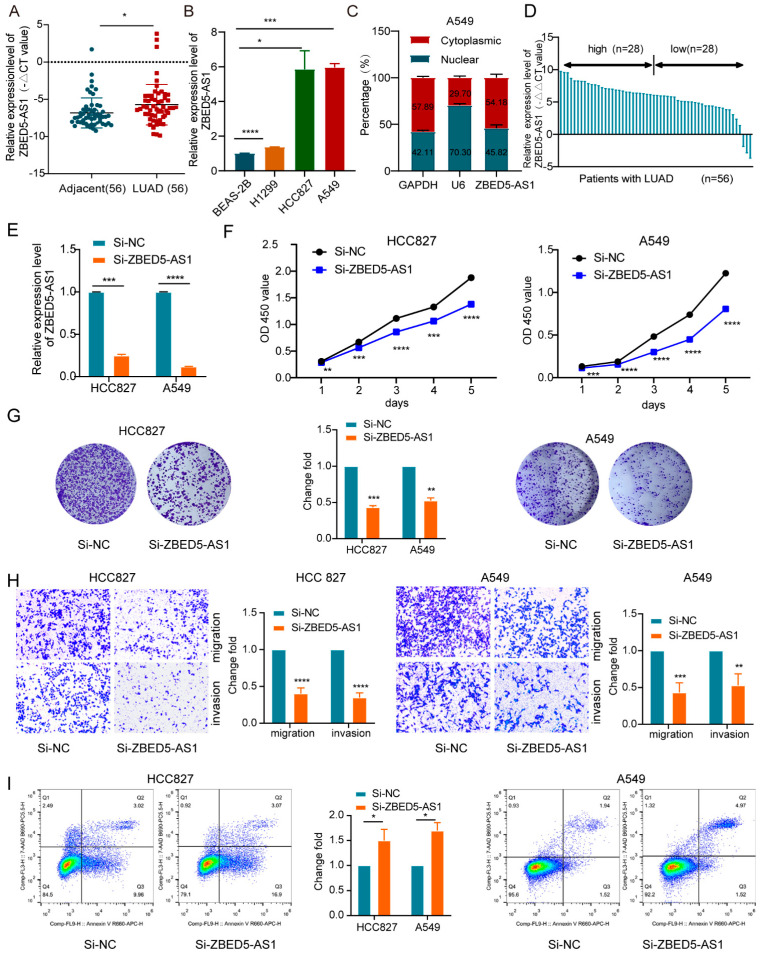
ZBED5-AS1 promotes LUAD cell proliferation, migration, and invasion in vitro and facilitates tumor growth in vivo. (**A**) ZBED5-AS1 expression was examined in 56 pairs of LUAD tissues and adjacent normal tissues by RT-qPCR. (**B**) Comparison of ZBED5-AS1 level in 4 cell types: BEAS-2B, H1299, A549, HCC827. (**C**) Subcellular fractionation was employed to determine the cellular localization of ZBED5-AS1 in A549 cells. (**D**) ZBED5-AS1 expression in LUAD and paracancerous tissues. (**E**) RT-qPCR was performed to determine Si-ZBED5-AS1 knockdown efficiency. (**F**,**G**) CCK-8 and Colony formation analysis was used to analyze the effect of ZBED5-AS1 knockdown on cell proliferation. (**H**) The invasive and migratory ability of LUAD cells with ZBED5-AS1 deficiency was detected by transwell invasion. (**I**) Flow cytometry was used to assess the cell apoptosis rate after ZBED5-AS1 knockdown. **** *p* < 0.0001, *** *p* < 0.001, ** *p* < 0.01, * *p* < 0.05.

We examined ZBED5-AS1 expression in LUAD patient samples via qRT-PCR and found that ZBED5-AS1 was expressed at significantly higher levels in 56 LUAD tissues than in their counterparts (*p* < 0.05; Figure 1A). Moreover, ZBED5-AS1 expression was upregulated in LUAD cells (H1299, HCC827, and A549) compared with BEAS-2B (Figure 1B). ZBED5-AS1 was distributed in both the cytoplasm (45.82%) and nucleus (54.18%), as confirmed by nuclear/cytoplasmic fractionation (Figure 1C). Subsequently, we conducted an analysis of the clinical data to investigate the clinical significance of ZBED5-AS1 expression in LUAD patients. On the basis of the median level of ZBED5-AS1, the 56 patients with LUAD were categorized into high (*n* = 28) and low (*n* = 28) expression groups (Figure 1D). We observed a significant association between ZBED5-AS1 expression and the elevated inflammatory marker LDH (Table 1). Furthermore, we discovered that increased coagulation marker D-dimer levels were linked to ZBED5-AS1 expression in LUAD patients (Table 1). However, there was no significant correlation between ZBED5-AS1 expression and age, tumor size, T stage, N stage, M stage, serum carcinoembryonic antigen (CEA), neuron-specific enolase (NSE), and progastrin-releasing peptide (ProGRP). Elevated serum LDH levels and a hypercoagulable state have been demonstrated to be indicative of a more aggressive disease [19,20]. These findings suggest that ZBED5-AS1 promotes the malignant phenotype of LUAD.

### 2.2. ZBED5-AS1 Promotes Malignant Phenotypes of LUAD Cells

To dissect the role of ZBED5-AS1 in LUAD, we constructed ZBED5-AS1 knockdown (Si-ZBED5-AS1) and overexpression (Oe-ZBED5-AS1) cells (Figure 1E and Figure S1A). To determine whether ZBED5-AS1 facilitates cell growth in LUAD cells, CCK-8 and colony formation assays were performed. The results indicated that ZBED5-AS1 depletion led to a decrease in LUAD cell proliferation (Figure 1F,G); however, Oe-ZBED5-AS1 showed an opposite trend (Figure S1B,C). The results indicate that ZBED5-AS1 considerably accelerated LUAD cell growth. Furthermore, the migration and invasion capabilities of LUAD cells were hindered significantly as a result of the knockdown of ZBED5-AS1, whereas ZBED5-AS1 overexpression upregulated the processes (Figure 1H and Figure S1D). The findings demonstrate that ZBED5-AS1 markedly accelerates cell migration and invasion in LUAD. Furthermore, we observed that silencing ZBED5-AS1 facilitated apoptosis, whereas ZBED5-AS1 overexpression had the opposite effect (Figure 1I and Figure S1E). The findings confirm that ZBED5-AS1 enhances aggressive characteristics in LUAD.

**Figure 2:**
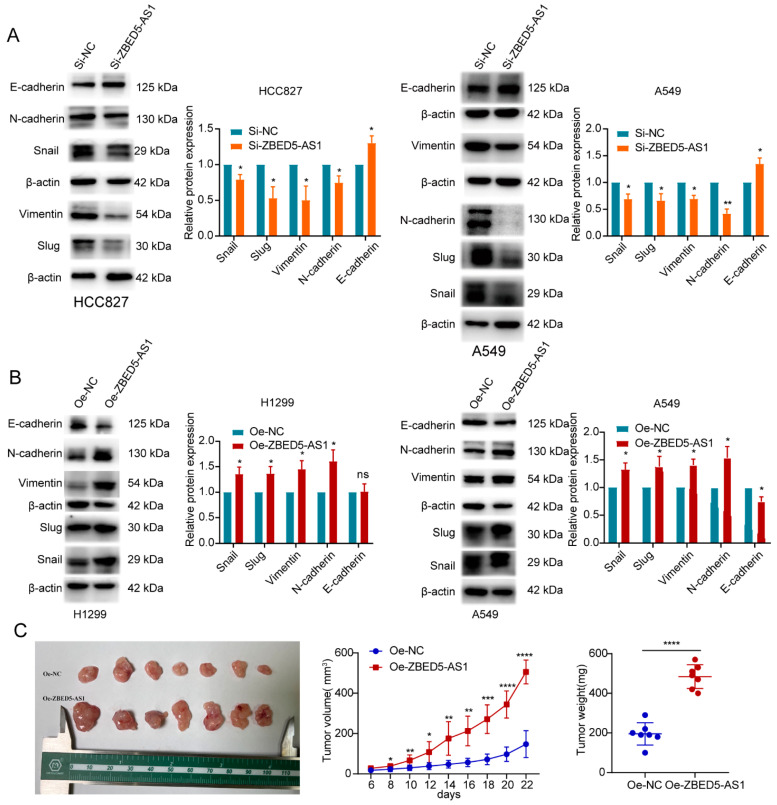
Western blot analysis of the epithelial–mesenchymal transition (EMT) markers and ZBED5-AS1 enhanced LUAD tumor growth in vivo. (**A**,**B**) The protein expression of EMT-related molecules by Western blot in ZBED5-AS1 overexpression and knockdown cells. (**C**) Animal experiments were conducted to evaluate ZBED5-AS1’s impacts on tumor growth. Representative images of tumors were displayed, and tumor volume and weights were also analyzed. The weight of tumors collected on day 24 and the volume of tumors measured every other day. **** *p* < 0.0001, *** *p* < 0.001, ** *p* < 0.01, * *p* < 0.05; ns: no significance.

Epithelial–mesenchymal transition (EMT) is a key step in tumor invasion and migration [21]. Therefore, we examined the effects of ZBED5-AS1 on the expression of EMT-related proteins. Compared with control cells, depletion of ZBED5-AS1 increased E-cadherin expression, accompanied by a downregulated expression of N-cadherin, Vimentin, Slug, and Snail (Figure 2A). In contrast, expression levels of N-cadherin, Vimentin, Slug, and Snail were significantly upregulated in LUAD cells overexpressing ZBED5-AS1 (Figure 2B). The findings indicated that ZBED5-AS1 can increase the migration and invasion ability of the cells as a result of EMT.

Mice were injected with A549 cells stably expressing empty vector or ZBED5-AS1. As shown in Figure 2C, when compared with the Oe-NC group, the tumor volume and weight in the Oe-ZBED5-AS1 group generally increased. All the findings above demonstrated that ZBED5-AS1 exacerbated tumor growth in vivo.

### 2.3. ZNF146 Enhances the Malignant Phenotypes of LUAD Cells

In order to tackle the mechanism whereby ZBED5-AS1 may contribute to the oncogenic response, RNA-seq was performed to compare the transcriptomes of the control groups and the cells with knockdown of ZBED5-AS1 (Figure S2A,B). Differential analysis was conducted on the expression data of A549 Si-ZBED5-AS1/A549 Si-NC groups, resulting in 249 differentially expressed genes, including 140 upregulated genes and 109 downregulated genes. Similarly, differential analysis was performed on the expression data of H1299 Si-ZBED5-AS1/H1299 Si-NC groups, yielding 206 differentially expressed genes, including 94 upregulated genes and 112 downregulated genes. The intersection of differentially expressed genes obtained from H1299 and A549 sequencing yielded 14 candidate genes (Figure S2C). Afterward, lung adenocarcinoma-related expression data and corresponding clinical information were downloaded from TCGA and HPA databases. The results showed that mRNA and protein levels of ZNF146 were highly expressed in lung adenocarcinoma (Figure S2C,D). Further analysis revealed that the AUC value of ZNF146 in tissue was 0.852, with a 95% CI of 0.82–0.885 (Figure S2E). Therefore, ZNF146 was chosen as the target gene for experimental research.

**Figure 3:**
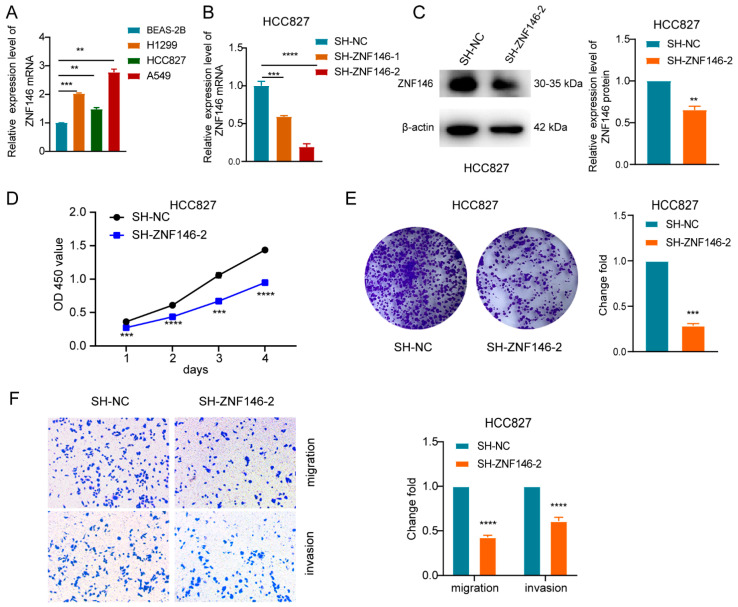
ZNF146 promotes LUAD cell proliferation, migration, and invasion. (**A**) Expression level of ZNF146 in LUAD cell lines and normal cell line BEAS-2B detected by qRT–PCR. (**B**,**C**) RT-qPCR and Western blot were performed to test the efficiency of ZNF146 knockdown in HCC827 cells. (**D**) Proliferative ability was examined via CCK-8 after indicated transfections in HCC827 cells. (**E**) Colony formation assays were used to analyze the effect of ZNF146 knockdown on cell proliferation in HCC827 cells. (**F**) Cell invasion and migration were analyzed through the implementation of transwell assay after transfection of indicated plasmids in HCC827 cells. **** *p* < 0.0001, *** *p* < 0.001, ** *p* < 0.01.

We found that ZNF146 was overexpressed in LUAD cells (Figure 3A). RT-qPCR and Western blot were performed to test the efficiency of ZNF146 knockdown in LUAD cells (Figure 3B,C and Figure S3A,B). The CCK-8 and colony formation assays revealed that ZNF146 knockdown suppressed the proliferation of LUAD cells (Figure 3D,E and Figure S3C,D). Furthermore, ZNF146 downregulation restricted the migration and invasion of LUAD cells (Figure 3F and Figure S3E). In summary, we found that ZNF146 plays an oncogenic role in LUAD cells.

### 2.4. ZNF146 Knockdown Inhibits LUAD Cell Proliferation and Invasion Mediated by ZBED5-AS1

**Figure 4:**
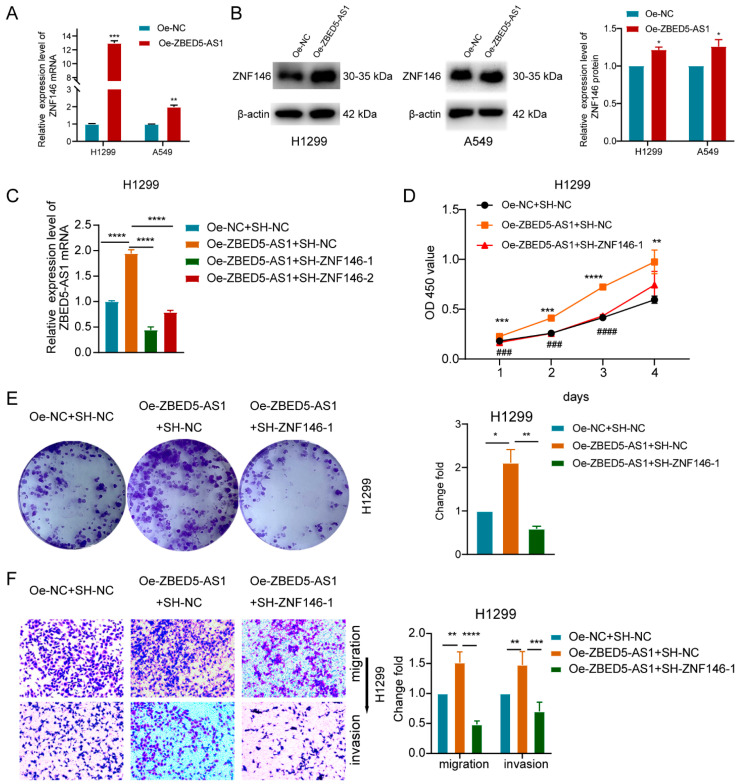
ZBED5-AS1 promotes the proliferation and invasion of LUAD cells by ZNF146. (**A**,**B**) ZNF146 expression was detected before and after ZBED5-AS1 overexpression in LUAD cells through RT-qPCR and Western blot. (**C**) ZNF146 expression after co-transfection of Oe-ZBED5-AS1 with SH-ZNF146 in H1299 cells by RT-qPCR. (**D**) CKK-8 assay analysis of LUAD cells proliferation after co-transfected with Oe-ZBED5-AS1 and SH-ZNF146 in H1299 cells. (**E**) The colony formation assay analysis of LUAD cells proliferation after co-transfected with Oe-ZBED5-AS1 and SH-ZNF146 in H1299 cells. (**F**) Migration assay and invasion assay analysis of LUAD cells migration effect of H1299 cells after co-transfected with Oe-ZBED5-AS1 and SH-ZNF146. **** *p* < 0.0001, *** *p* < 0.001, ** *p* < 0.01, * *p* < 0.05, ^####^ *p* < 0.0001, ^###^ *p* < 0.001.

We further investigated whether ZNF146 was the downstream effector of ZBED5-AS1 and found that ZBED5-AS1 overexpression was associated with increased mRNA and protein levels of ZNF146, whereas its knockdown had the opposite effects (Figure 4A,B and Figure S4A,B). To investigate whether ZNF146 is involved in ZBED5-AS1-mediated proliferation and invasion, Si-ZNF146 and Oe-ZBED5-AS1 were co-transfected into H1299 and HCC827 cells. Co-transfection with Si-ZNF146 notably decreased ZBED5-AS1-induced upregulation of ZBED5-AS1 expression (Figure 4C and Figure S5A). A series of in vitro rescue experiments showed that ZBED5-AS1 overexpression and simultaneous suppression of ZNF146 resulted in reduced proliferation, migration, and invasion capacities (Figure 4D–F and Figure S5B–D). According to the results, the ZBED5-AS1-induced proliferation, invasion, and migration of LUAD cells can be reversed by ZNF146 expression.

### 2.5. ZBED5-AS1 Silencing Suppresses the Activation of ATR/Chk1 Signaling Pathway

**Figure 5:**
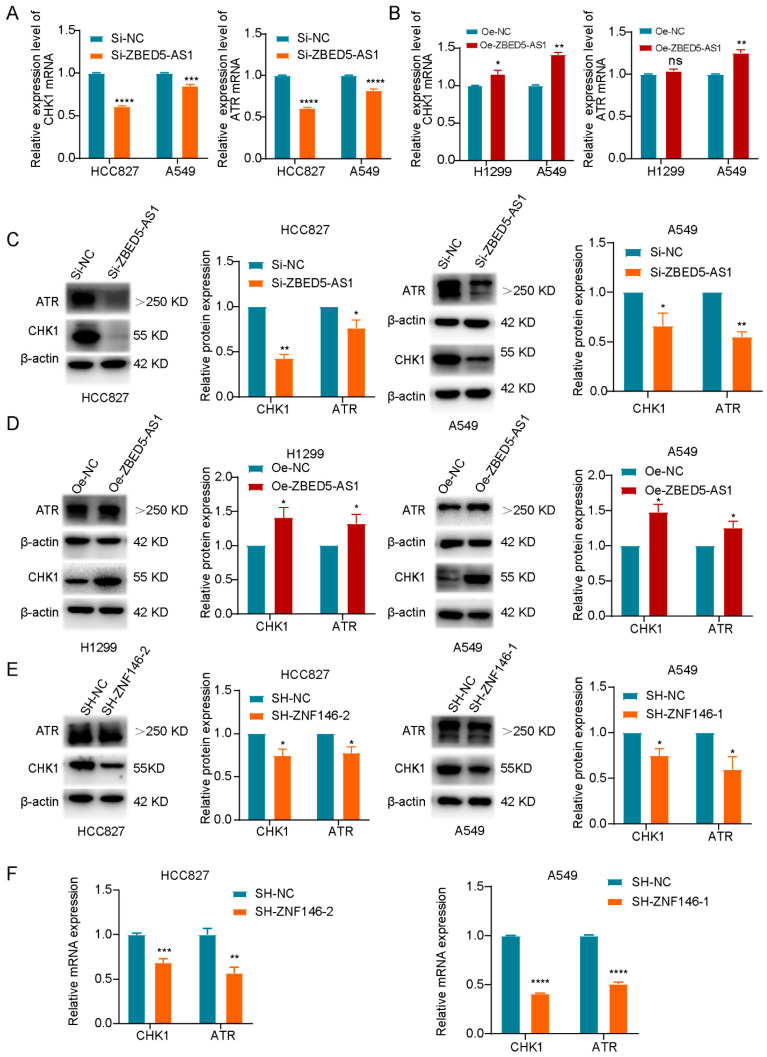
ZBED5-AS1 activates the ATR/Chk1 pathway. (**A**,**B**) The changes in ATR and Chk1 mRNA levels in cells transfected with Si-ZBED5-AS1 or Oe-ZBED5-AS1 were assessed via RT-qPCR. (**C**,**D**) The changes in ATR and Chk1 protein levels in cells transfected with Si-ZBED5-AS1 or Oe-ZBED5-AS1 were assessed via Western blot. (**E**) The influence of ZNF146 silence on ATR and Chk1 mRNA levels was evaluated via RT-qPCR. (**F**) The influence of ZNF146 silence on ATR and Chk1 protein levels was evaluated via Western blot. **** *p* < 0.0001, *** *p* < 0.001, ** *p* < 0.01, * *p* < 0.05.

It is widely recognized that the ATR/CHK1 signaling pathway is commonly upregulated in cancer and promotes tumor growth. Inhibition of this pathway has been shown to suppress cell apoptosis, invasion, and migration, ultimately leading to the elimination of tumor cells [16]. Therefore, further investigations were conducted to elucidate the impact of ZBED5-AS1 expression on the ATR/Chk1 signaling pathway. RT-qPCR and Western blot analyses were performed to assess the effect of ZBED5-AS1 on the activation of the ATR/Chk1 signaling pathway-related genes, ATR and Chk1. In the Si-ZBED5-AS1 cell line, the expression of ZBED5-AS1, ATR, and Chk1 was decreased compared with that in the NC groups. In contrast, in the Oe-ZBED5-AS1 group, the expression of ZBED5-AS1 was upregulated (Figure 5A–D). The results suggest that ZBED5-AS1 promotes the activation of the ATR/Chk1 pathway. Moreover, ZNF146 knockdown downregulated the mRNA and protein levels of ATR and Chk1 in LUAD cells (Figure 5E,F). These results verified that ZBED5-AS1 could enhance ATR/Chk1 pathway activity.

### 2.6. Si-ZBED5-AS1 Exosomes Inhibite LUAD Cells Proliferation and Metastasis

**Figure 6:**
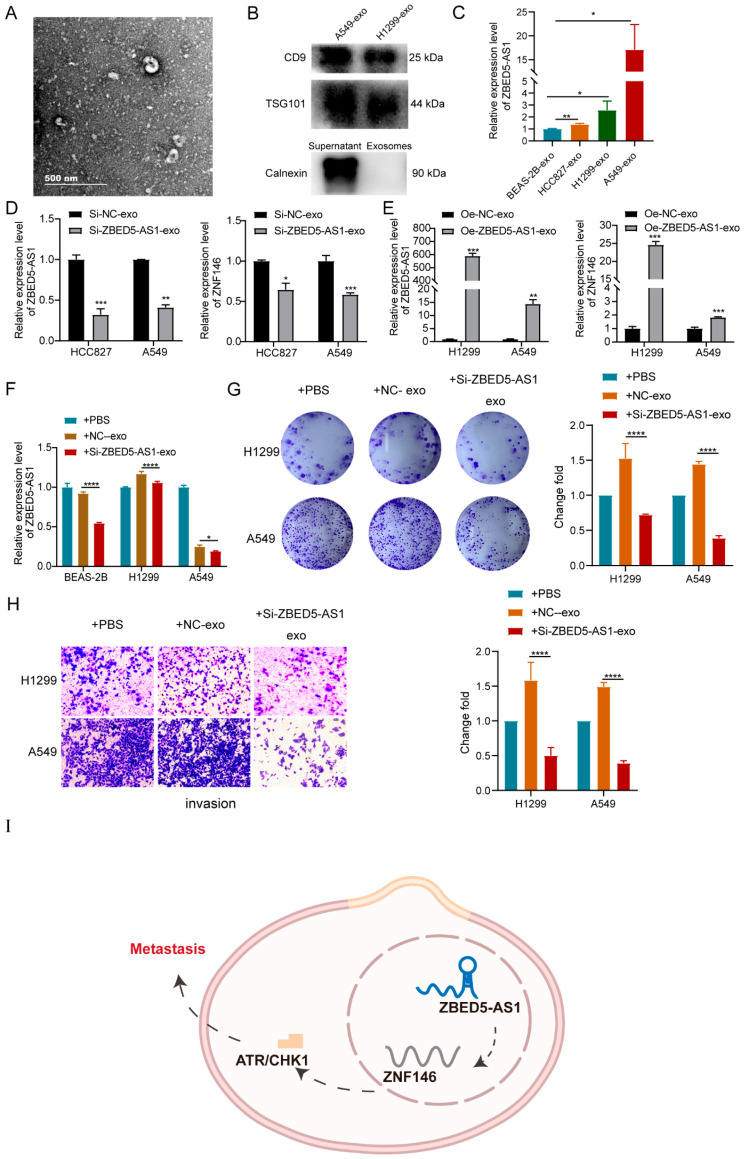
Si-ZBED5-AS1 exosomes inhibited LUAD cells proliferation and metastasis. (**A**) The concentration and size distribution were determined by nanoparticle tracking analysis (NTA). (**B**) A549- and H1299-exosomes were analyzed by Western blot for the presence of the exosomal protein markers CD9 and TSG101. Additionally, the levels of calnexin (an exosome-negative marker) were measured in the “supernatant” of A549 cells and the exosome fraction. The “supernatant” represents the final supernatant obtained during the exosome extraction process. (**C**) Exosomal ZBED5-AS1 expression in BEAS-2B cells and LUAD cell lines was analyzed by RT-qPCR. β-actin was used as a loading control. (**D**) The mRNA level of ZBED5-AS1 and ZNF146 in ZBED5-AS1 knockdown and its control cells exosomes. (**E**) The mRNA level of ZBED5-AS1 and ZNF146 in ZBED5-AS1 overexpression and its control cells exosomes. (**F**) ZBED5-AS1 expression after coculturing with Si-NC or Si-ZBED5-AS1 cell-derived exosomes from HCC827 cells. (**G**) The colony formation assays detected the clone formation ability of H1299 and A549 cells after coculturing with exosomes derived from ZBED5-AS1 knockdown. (**H**) H1299 and A549 cells were treated with exosomal ZBED5-AS1knockdown, and the invasion capabilities were evaluated by transwell assay. (**I**) Schematic illustration of our proposed model. ZBED5-AS1 enhanced the metastatic potential of LUAD cells through the EMT pathway by activating ZNF146/ATR/CHK1 axis. **** *p* < 0.0001, *** *p* < 0.001, ** *p* < 0.01, * *p* < 0.05.

We extracted exosomes from the culture media of A549, H1299, HCC827, and BEAS-2B cells and subjected them to TEM analysis (Figure 6A). With the typical enrichment of exosome marker proteins (CD9 and TSG101) and the absence of calnexin, confirmation was obtained regarding the successful extraction of exosomes (Figure 6B). Furthermore, an observation was made that ZBED5-AS1 exhibited higher expression levels in exosomes derived from LUAD cell lines compared with those derived from BEAS-2B cells. ZBED5-AS1 and ZNF146 expression decreased in Si-ZBED5-AS1 exosomes but increased in Oe-ZBED5-AS1 exosomes (Figure 6D,E). Next, we isolated Si-NC or Si-ZBED5-AS1 cell-derived exosomes from HCC827 cells. These isolated exosomes were then cocultured with BEAS-2B, H1299, and A549 cells. The subsequent results demonstrated a decrease in the expression of ZBED5-AS1 in BEAS-2B, H460, and A549 cells that were cocultured with Si-ZBED5-AS1 exosomes resulted in reduced expression of ZBED5-AS1 in LUAD cells (Figure 6F). Moreover, colony formation assay and transwell assay showed that the proliferation and invasion viability of LUAD cells that were cocultured with Si-ZBED5-AS1-exosome were lower than those in LUAD cells that were cocultured with Si-NC-exosome (Figure 6G,H). The results indicate that Si-ZBED5-AS1 exosomes can be delivered into LUAD cells, leading to a reduction in LUAD cells proliferation and metastasis.

## 3. Discussion

Recent studies have shown that several novel lncRNAs are potentially involved in LUAD oncogenesis and progression through multiple mechanisms [22]. For instance, Wang et al. suggested that FAM83H-AS1 promotes LUAD development by binding heterogeneous nuclear ribonucleoprotein K and increasing the expression of antiapoptotic oncogenes RAB8B/ RAB14 [23]. Furthermore, Zhang et al. demonstrated that lncRNA SNHG17 induces LATS2 expression and promotes LUAD gefitinib resistance [24]. Therefore, the identification and research on LUAD-associated lncRNAs is essential for understanding the roles of lncRNAs in tumorigenesis and improving the current clinical setting. The exact role and mechanisms of function of ZBED5-AS1 in LUAD have not been reported previously. In the present study, significant overexpression of ZBED5-AS1 was observed in LUAD tissues and cells. Furthermore, we observed that ZBED5-AS1 is located mainly in the cytoplasm of LUAD cells. ZBED5-AS1 was found to promote the proliferation, migration, and invasion of LUAD cells and inhibit their apoptosis. This outcome is in line with our previous finding that ZBED5-AS1 exerts oncogenic effects on LUAD progression [9]. Additionally, EMT has been widely acknowledged as a crucial mechanism in the development of tumors. During EMT, a polarized epithelial cell undergoes a phenotypic transition into a mesenchymal cell, exhibiting heightened migratory and invasive capabilities, as well as resistance to apoptosis [25]. It has been increasingly recognized that various factors are involved in the process of EMT, particularly noncoding RNAs, which play a crucial role in regulating EMT and tumor invasion and metastasis [3]. Here, we found that ZBED5-AS1 upregulated the expression of EMT-associated markers, leading to enhanced migration and invasion abilities of LUAD cells.

Tumor metastasis is known to cause an increase in LDH levels [26]. In addition, there has been a growing focus on the relationship between malignancy progression and coagulation [27]. A more aggressive disease can be indicated by hypercoagulability, which is a disturbance of hemostasis characterized by elevated D-dimer levels [20]. In our study, we have discovered that elevated LDH and D-dimer were positively correlated with ZBED5-AS1. These findings further demonstrated that ZBED5-AS1 played an oncogenic role in LUAD and may be associated with tumor metastasis. Given the limited sample size in our study, we observed no significant association between the expression of ZBED5-AS1 and T stages, N stages, or M stages. Through RNA-seq screening and subsequent validation via transcriptional and functional assays, it has been discovered that increased ZNF146 levels play a critical role in oncogenic response. Moreover, the downregulation of ZNF146 reversed the malignant phenotype of overexpressing ZBED5-AS1 on cell proliferation, invasion, and migration. In fact, similar to our discovery, ZNF146 upregulation has been well-documented in hepatocellular carcinoma and colorectal cancer [11,28,29]. In addition, ZNF146 was observed to be the downstream target of the LncRNA KCNQ1OT1, and it promoted the development of colorectal cancer [29]. In addition, ZNF146 was overexpressed in gastric cancer tissues and regulated the progression of gastric cancer through the CircPIP5K1A [10]. Here, we uncovered a novel signal axis where ZBED5-AS1 promoted the progression of LUAD via modulation of the expression of ZNF146.

ATR belongs to the phosphatidylinositol 3-kinase-related kinase (PIKK) family called serine/threonine-protein kinase. ATR is also a crucial player in DNA replication stress response and DNA-damage-activated checkpoints [30]. In response to an activating signal, such as single- and double-stranded DNA, inhibited and cross-linked DNA polymerase, the level of ATR increases [31]. ATR is located upstream of Chk1, and its direct substrates include p53, RPA, MCM2, and many other factors that play roles in DNA repair, replication fork progression, and cell cycle [32,33]. A recent study showed that Chk1 mRNA encoding Chk1 was significantly overexpressed in small-cell lung cancer compared with non-small cell lung cancer samples. It was further shown that Chk1 and ATR inhibition could induce genotoxic stress response and subsequent apoptosis, especially in small-cell lung cancer cells, whereas non-small cell lung cancer cells exhibited resistance to ATR/Chk1 inhibition [34]. Hence, the ATR/Chk1 pathway is a novel pathway that could be explored for anticancer therapy development in small-cell lung cancer. In addition, some findings of the present study suggest that ZBED5-AS1 activated the ATR/ Chk1 signaling pathway in LUAD.

Exosome-mediated intercellular communication has been studied extensively [35]. Notably, lncRNAs are the key components of tumor cell-derived exosomes [36,37]. Exosomal lncRNAs are involved in the proliferation, drug resistance, and metastasis of various cancers and exert their biological effects directly in the recipient cells [38]. We discovered that exosomes from LUAD cells have higher expression of ZBED5-AS1 than exosomes from normal cell line BEAS-2B. Moreover, exosomes derived from Si-ZBED5-AS1 cells had lower levels of ZBED5-AS1 compared with exosomes derived from Si-NC cells. Further investigation confirmed that Si-ZBED5-AS1 exosomes decreased LUAD cells proliferation and invasion. In conclusion, to the best of our knowledge, this is the first study to show that exosomes with lower ZBED5-AS1 levels produced lower efficiency in the proliferation and metastasis of LUAD cells. However, the specific regulatory mechanism of exosome-derived ZBED5-AS1 and ZNF146/ATR/Chk1 axis in LUAD progression needs to be studied further. Serum exosomes are promising biomarkers for circulatory diseases in LUAD [39], and since ZBED5-AS1 was associated with LUAD metastasis in our study, the number of metastatic patients will be increased in future studies. The correlation between the expression of tissue ZBED5-AS1 and serum exosome ZBED5-AS1 and metastasis was analyzed, and whether it could be used as a biomarker for LUAD metastasis was evaluated.

The current study indicates that ZBED5-AS1 could be a key promoter of LUAD metastasis through the EMT pathway by activating ZNF146/ATR/CHK1 axis (Figure 6I). Taken together, these discoveries might provide new possibilities for treating LUAD, especially for cases with metastasis.

## 4. Materials and Methods

### 4.1. Patient Samples

LUAD tumors and adjacent noncancerous lungs were collected from 56 patients (26 male and 30 female) who underwent routine surgery between 2017 and 2020 at the First Affiliated Hospital of Wenzhou Medical University (Zhejiang, China). None of the LUAD patients received adjuvant therapy before participating in this study. All clinical procedures were approved by the Committee for Ethical Review of the First Affiliated Hospital of the Wenzhou Medical University (WYDW2020-0380).

### 4.2. Animal Experiments

Six-week-old BALB/c nude mice (Zhejiang Weitong Lihua Laboratory Animal Technology Co. Ltd., Hangzhou, China) were used to induce allograft tumor growth. All animal experiments were conducted after obtaining approval from the Laboratory Animal Ethics Committee of the First Affiliated Hospital of the Wenzhou Medical University. Mice were subdivided randomly into Si-NC (*n* = 6) or Si-ZBED5-AS1 (*n* = 6) groups. Transfected cells (5 × 10^6^) were injected subcutaneously into mice. After the tumors formed, the pulmonary tumor volume and weight of the mice were recorded every two days. The volume was calculated using the formula (length × width^2^)/2. The mice were sacrificed on day 24 via excessive anesthesia, and the tumors were resected, photographed, and weighed.

### 4.3. Cell Culture

Four LUAD cell lines were involved in this work: A549, HCC827, and H1299, and the lung epithelial cell line BEAS-2B. All the cells above were obtained from the Cell Bank of the Chinese Academy of Sciences (Shanghai, China) and were cultured in RPMI-1640 medium (Gibco, Waltham, MA, USA) containing 10% fetal bovine serum (FBS, Gibco). The cells were incubated at 37 °C in a sterile humidified CO_2_ (5%) incubator.

### 4.4. Cell Transfection

Si-ZBED5-AS1 (silenced ZBED5-AS1), Si-NC (control for the silenced group), Oe-ZBED5-AS1 (overexpressed ZBED5-AS1), Oe-NC (control for ZBED5-AS1 overexpression), and SH-ZNF146 (silenced ZNF146) were synthesized by Genechem Company (Shanghai, China). Transfection of all constructed lentiviruses was performed using Lipofectamine 3000 (Life Technologies, Carlsbad, CA, USA). Subsequently, 50 nM siRNAs were transfected into LUAD cells with Lipofectamine 3000 (Invitrogen, Shanghai, China). Transfection reactions were promoted using an Opti-MEM medium (Gibco). After 10-h transfection, the medium was changed into a complete RPMI-1640 medium. Stably transfected cells were selected using puromycin (2 μg/mL) (Solarbio, Beijing, China) after 3 days.

### 4.5. Subcellular Fractionation

Cytoplasmic and nuclear RNA fractions were isolated from A549 and H1299 cells using a nuclear/cytosol fractionation kit (BioVision, Milpitas, CA, USA) to localize ZBED5-AS1. Specific RNA extraction was performed with TRIzol reagent, after which the expression levels in the cytoplasm and nucleus were assessed via RT-qPCR. U6 and GAPDH served as markers of nuclear and cytoplasmic controls, respectively.

### 4.6. Exosome Isolation and Identification

Before exosome isolation, LUAD cells were cultured in a medium supplemented with exosomal-free FBS for 48 h. Cell culture supernatants were collected, and exosomes were obtained by differential centrifugation at 500× *g* for 8 min, 2000× *g* for 10 min, and 12,000× *g* for 30 min to separate the floating and detached cells. The supernatant was then passed through syringe filters (0.22-μm pore size) (Millipore, USA), followed by ultracentrifugation at 120,000× *g* for 2 h to isolate the exosomes and a wash with PBS. All centrifugation processes were carried out at 4 °C. The pellet was either used immediately or stored at −80 °C. The Bicinchoninic Acid (BCA) test (Thermo Fisher Scientific, Waltham, MA, USA) was used to quantify protein concentrations of the exosomes.

Approximately 10 μL of the exosome solution was dropped on a carbon film copper mesh, incubated at room temperature, and air-dried for 10 min. Exosomes were then stained with 50 μL of 3% phosphotungstic acid solution for 5 min at room temperature. The grids were air-dried for 5 min, and the images were captured using a transmission electron microscope (TEM; Hitachi, Tokyo, Japan). The total amounts of exosomes were determined by nanoparticle tracking analysis (NTA) using Nanosight NTA NS300 (Malvern Instruments Ltd., Malvern, UK). Exosome protein markers (CD9, TSG101, and Calnexin) were confirmed by Western blotting, and β-actin was used as a loading control.

### 4.7. RNA Extraction and RT-qPCR

Total RNA was isolated from tissues, cells, and exosomes using TRIzol reagent (Invitrogen, CA, USA). cDNA was synthesized from 0.5 ug total RNA using a cDNA synthesis kit (Takara, Kusatsu, Japan). Subsequently, a qRT-PCR Kit (Takara) was used for RT-qPCR analysis on an ABI 7500 (Applied Biosystems, Foster City, CA, USA) real-time PCR machine. For mRNA quantification, β-actin was used as an endogenous control, whereas U6 was used for lncRNA quantification. The 2^−ΔΔCt^ method was used for calculating the relative changes in expression. Specific PCR primers (Table S1) were supplied by Sangon Biotech (Shanghai, China).

### 4.8. Exosome Treatment

Si-NC or Si-ZBED5-AS1 cell-derived exosomes from HCC827 cells were isolated from a 50-mL medium containing 1 × 10^7^ cells. When the cells reached approximately 70% confluence, H1299 and A549 cells were directly cultured with 50 μg/mL exosomes-ZBED5-AS1 or PBS as a control [40,41]. Cells were collected for analysis 48 h after treatment. 

### 4.9. Cell Proliferation Assay

Cells were plated in 96-well plates (4 × 10^3^ cells/well), and their proliferation was evaluated using a Cell Counting Kit-8 (CCK-8, Dojindo, Japan). Briefly, CCK-8 solution (10 μL/well) was added and incubated for 1 h at 37 °C, after which the absorbance was recorded using a microplate reader at 450 nm (SpectraMax, MD, USA). The CCK-8 assays were performed for four consecutive days.

For the colony formation assay, transfected cells (5 × 10^2^ cells/well) were seeded into 6-well plates containing 2 mL medium and maintained for 14 days. The medium was refreshed every four days. Colonies were imaged and counted after they were fixed with 4% paraformaldehyde for 30 min and dyed with 0.2% crystal violet (Beyotime, Shanghai, China) for 15 min.

### 4.10. Transwell Assay

Transwell migration and invasion assays were carried out in Transwell-24 well plates containing 8-um pore filters. (Corning, Tewksbury, MA, USA). Serum-free medium with transfected LUAD cells (4 × 10^4^ cells/100 µL) was plated into the upper chamber, while 20% FBS was seeded into the lower chamber. A Matrigel-coated membrane (Corning) was placed on the surface of the upper chamber for the invasion assay. After 48-h incubation, the invading cells passed through the membrane. The invading cells were fixed with 4% paraformaldehyde and stained with 0.2% crystal violet.

### 4.11. Flow Cytometry

Transfected cells (2.0 × 10^5^) were plated into 6-well plates and stained using an apoptosis detection kit (KeyGEN, Nanjing, China). Briefly, after washing twice with ice-cold PBS, cells were resuspended in 500 μL binding buffer and stained with 5 μL Annexin-V-APC and 5 μL 7-AAD staining solution for 10 min in the dark. The cells were analyzed using a flow cytometer (BD FACSCalibur, San Diego, CA, USA).

### 4.12. Western Blot

Total proteins from transfected cells were extracted with RIPA buffer (Beyotime) containing 1% protease inhibitor phenylmethylsulfonyl fluoride (PMSF, Beyotime) and then quantified using a BCA protein quantification kit (Beyotime). Subsequently, 20-μg samples of denatured proteins were fractioned by performing sodium dodecyl sulfate polyacrylamide gel electrophoresis (SDS-PAGE) using 10% resolving gels. The resolved proteins were transferred to polyvinylidene fluoride membranes (Merck Millipore, MA, USA). Upon blocking with 5% skimmed milk in 1× TBST for 2 h, membranes were incubated with the following primary antibodies overnight at 4 °C: Anti-E-cadherin (1:5000, Protiontech), Anti-N-cadherin (1:2000, Protiontech, Rosemont, IL, USA), Anti-Vimentin (1:2000, Protiontech), Anti-Slug (1:800, Protiontech), Anti-Snail (1:800, Protiontech), Anti-β-actin (1:1000, Protiontech), Anti-ZNF146 (1:1000, Protiontech), Anti-ATR (1:500, Protiontech), Anti-Chk1 (1:2000, Protiontech), Anti-Calnexin (1:40,000, Protiontech), Anti-TSG101 (1:2000, Protiontech), and Anti-CD9 (1:500, Protiontech). After washing off the primary antibody, the secondary antibody (1:2000, Beyotime) was dropped and incubated for 1 h at room temperature. Signals were captured using an enhanced chemiluminescence (ECL) substrate (Beyotime) and then imaged on an ECL imaging system (Bio-Rad, Hercules, CA, USA).

### 4.13. Statistical Analysis

IBM SPSS Statistics 22 (IBM Corp., Armonk, NY, USA) and R were used for statistical analysis. Each experiment was repeated at least three times, and the collected data are presented as mean ± standard deviation (SD). One-way analysis of variance (one-way ANOVA), Student’s *t*-test, Wilcoxon rank sum test, Fisher’s exact test, and Pearson Chi-square test were used to compare the changes between groups. The statistical significance was set at *p* < 0.05.

## Figures and Tables

**Table 1:** Correlation of ZBED5-AS1 expression with clinical characteristics.

Characteristics	Low Expression	High Expression	*p* Value
*n*	28	28	
gender, *n* (%)			1.0000
female	15 (26.8%)	15 (26.8%)	
male	13 (23.2%)	13 (23.2%)	
age, *n* (%)			0.5842
≤65	18 (32.1%)	16 (28.6%)	
>65	10 (17.9%)	12 (21.4%)	
T, *n* (%)			0.3881
T1	22 (39.3%)	21 (37.5%)	
T2	4 (7.1%)	5 (8.9%)	
T4	2 (3.6%)	0 (0%)	
T3	0 (0%)	1 (1.8%)	
X	0 (0%)	1 (1.8%)	
N.stage, *n* (%)			0.0860
N0	22 (39.3%)	26 (46.4%)	
N1	6 (10.7%)	1 (1.8%)	
X	0 (0%)	1 (1.8%)	
M.stage, *n* (%)			0.9999
M0	27 (48.2%)	27 (48.2%)	
M1	1 (1.8%)	0 (0%)	
X	0 (0%)	1 (1.8%)	
Tumor size, *n* (%)			0.1764
>2	9 (16.1%)	14 (25%)	
≤2	19 (33.9%)	13 (23.2%)	
X	0 (0%)	1 (1.8%)	
CEA (μg/L), median (IQR)	2.4 (1.35, 4.75)	2.45 (1.9, 6.3)	0.4775
NSE (ng/mL), median (IQR)	12.6 (9.95, 19.45)	13.1 (11, 16.2)	0.6602
ProGRP (ng/L), mean ± sd	48.708 ± 14.977	51.05 ± 23.442	0.7021
D-dimer, median (IQR)	0.345 (0.3, 0.72)	0.91 (0.37, 1.06)	0.0245
LDH (U/L), median (IQR)	178 (162, 189.5)	187 (178.5, 236)	0.0480
PLT counts, median (IQR)	231.5 (185.5, 276.5)	253 (222.5, 294)	0.2158

The median expression level of ZBED5-AS1 was used as the cutoff value. “X” indicated that the tumor could not be evaluated or measured for clinical characteristics, and these patients were not included in the statistical test. Abbreviation: CEA—carcinoembryonic antigen; NSE—neuron-specific enolase; ProGRP—progastrin-releasing peptide; LDH—lactate dehydrogenase; PLT—platelets.

## Data Availability

The data sets used and analyzed during the current study are available from the corresponding author upon reasonable request.

## Supplementary Materials

The supporting information can be downloaded at: https://www.mdpi.com/article/10.3390/ijms241813925/s1.

## Abbreviations

lncRNA—Long non-coding RNA; LUAD—lung adenocarcinoma; ZNF146—Zinc finger protein 146; FBS—fetal bovine serum; qPCR—quantitative polymerase chain reaction; CCK-8—cell counting kit-8; SDS-PAGE—sodium dodecyl sulfate polyacrylamide gel electrophoresis; PMSF—protease inhibitor phenylmethylsulfonyl fluoride; ECL—enhanced chemiluminescence; EMT—Epithelial–mesenchymal transition; TEM—transmission electron microscope; NTA—nanoparticle tracking analysis; SPSS—Statistical Product and Service Solutions.
