# Supplementary material for: Long Noncoding RNA ZBED5-AS1 Facilitates Tumor Progression and Metastasis in Lung Adenocarcinoma via ZNF146/ATR/Chk1 Axis
Source: Int J Mol Sci. 2023 Sep 10;24(18):13925. doi: 10.3390/ijms241813925 (PMC10530271; doi:10.3390/ijms241813925)
Supplement: Supplementary file 1 [file ijms-24-13925-s001.zip › Supplementary Methods and Figures.pdf]

Supplementary Methods:

Methods

RNA Sequencing Data Analysis

The total RNA of four samples (A549 Si-ZBED5-AS1, A549 Si-NC, H1299 Si-ZBED5-AS1, H1299 Si-NC) was isolated and extracted using TRIzol (Invitrogen, CA, USA) according to the manufacturer's instructions. The quantity and purity of the total RNA were assessed using NanoDrop ND-1000 (NanoDrop, Wilmington, DE, USA). The integrity of the RNA was checked using Bioanalyzer 2100 (Agilent, CA, USA) and validated by gel electrophoresis. Construct the library using the TruSeq Stranded mRNA LT Sample Prep Kit (Illumina, San Diego, CA, USA) according to the manufacturer's instructions. Finally, the library was sequenced using Illumina Novaseq™ 6000 (LC Bio Technology CO., Ltd. Hangzhou, China) in PE150 mode according to standard procedures. we performed adapter trimming, low-quality sequence filtering, and duplicate sequence removal on the raw data to obtain high-quality CleanData. We used HISAT2 software to align CleanData to the human genome and generated BAM files. Then, we conducted initial transcriptome assembly using StringTie and compared the transcripts with reference annotations using gffcompare to obtain the final assembly annotation results. FPKM quantification was performed using the ballgown package. Significant differential analysis of samples was conducted using DESeq2, with differentially expressed genes defined as those with a fold change (FC) greater than 2 or less than 0.5 and a p-value less than 0.05.

Supplementary Figures:

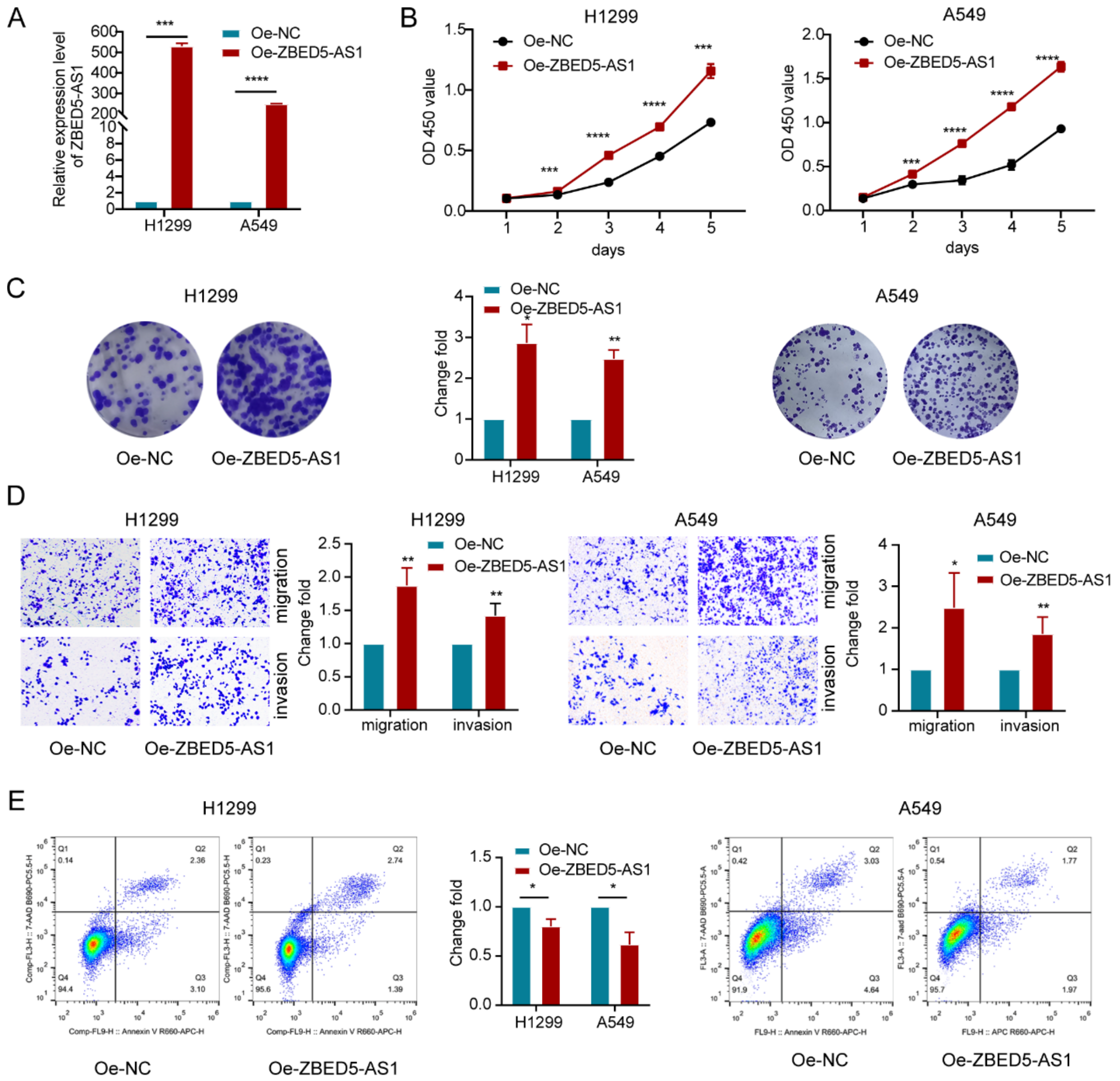

Figure S1. ZBED5-AS1 promotes LUAD cell proliferation, migration, and invasion in vitro and facilitates tumor growth in vivo. (**A**) RT-qPCR was done to determine Oe- ZBED5-AS1 overexpression efficiency. (**B**, **C**) CCK-8 and Colony formation analysis were used to analyze the effect of ZBED5-AS1 overexpression on cell proliferation. (**D**) Invasive and migratory ability of LUAD cells with ZBED5-AS1 overexpression was detected by transwell invasion. (**E**) Flow cytometry was used to assess the cell apoptosis rate after ZBED5-AS1 overexpression. **** $P<0.0001$, *** $P<0.001$, ** $P<0.01$, * $P<0.05$.

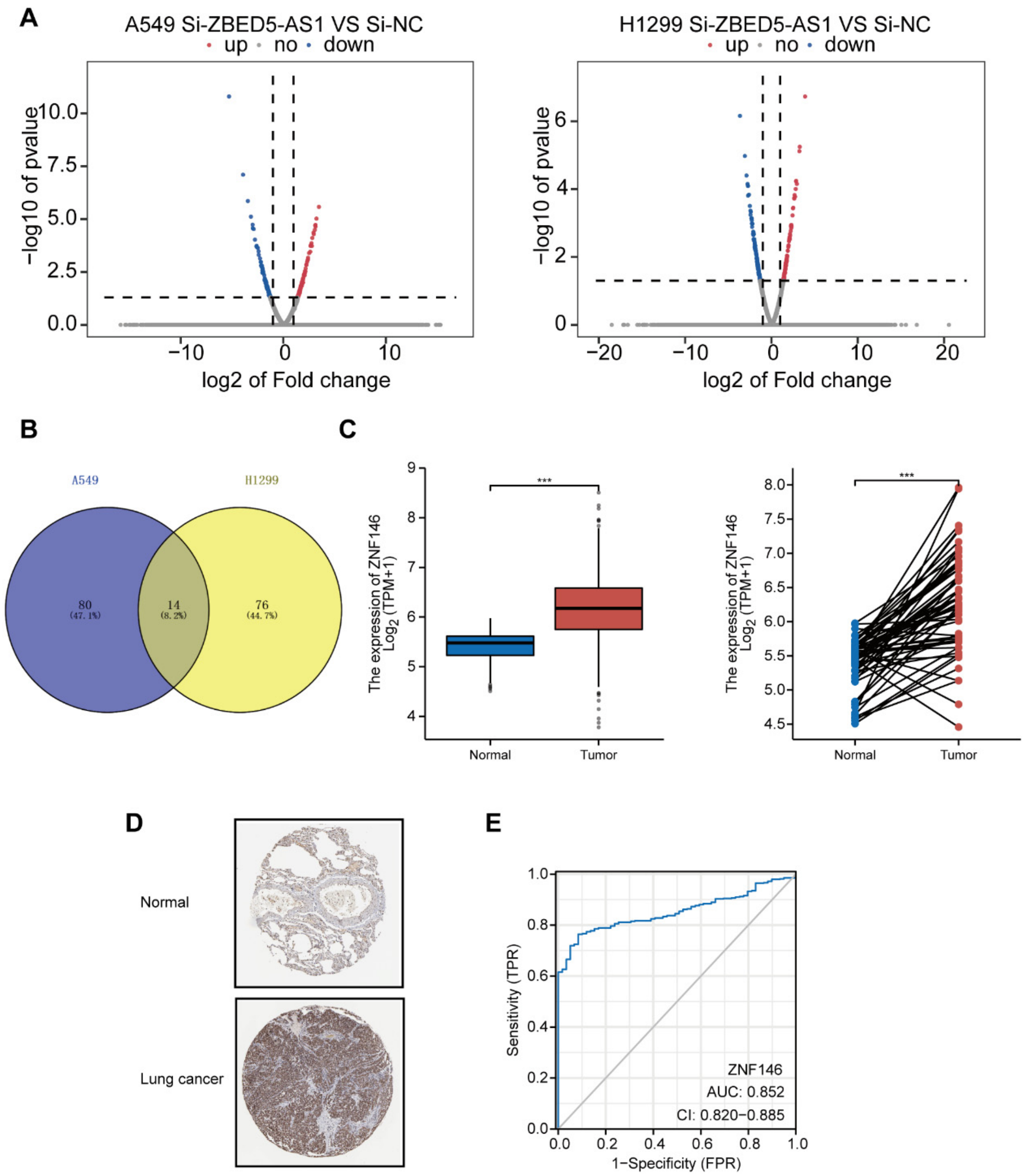

Figure S2. Screening of target genes. (**A**) Volcano plot. (**B**) Intersection of differential genes. (**C**) Expression of ZNF146 mRNA in TCGA database. (**D**) Expression level of ZNF146 protein in HPA database. (**E**) ROC curve of ZNF146. *** P<0.001.

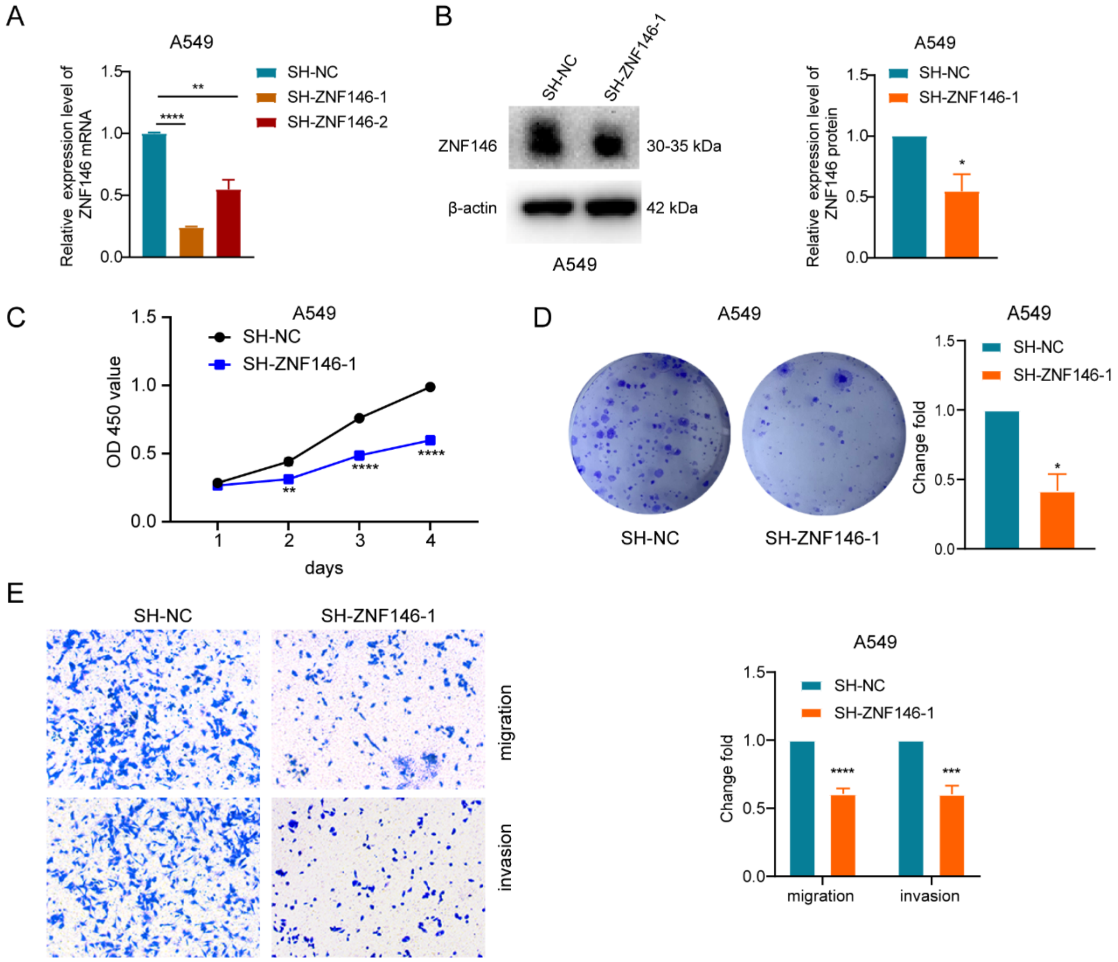

Figure S3. ZNF146 promotes LUAD cell proliferation, migration and invasion. (**A**, **B**) RT-qPCR and Western blot were performed to test the efficiency of ZNF146 knockdown in A549 cells. (**C**) Proliferative ability was examined via CCK-8 after indicated transfections in A549 cells. (**D**)Colony formation assays were used to analyze the effect of ZNF146 knockdown on cell proliferation in A549 cells. (**E**) Cell invasion and migration were analyzed through implementation of transwell assay after transfection of indicated plasmids in A549 cells. **** P<0.0001, *** P<0.001, ** P<0.01, * P<0.05.

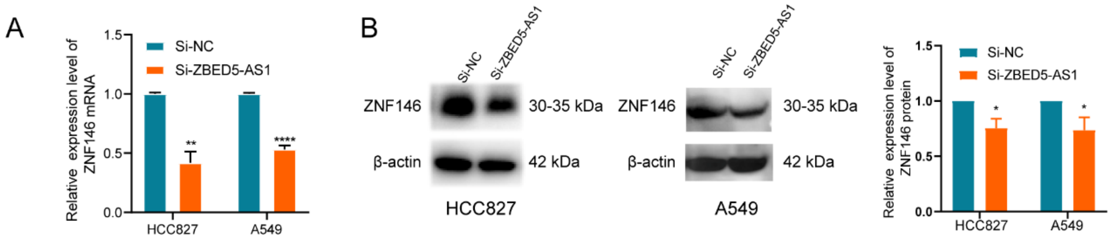

Figure S4. ZBED5-AS1 promotes the proliferation and invasion of LUAD cells by ZNF146. (**A**) ZNF146 expression was detected before and after ZBED5-AS1 downregulation in LUAD cells through RT-qPCR. (**B**)ZNF146 expression was detected before and after ZBED5-AS1 downregulation in LUAD cells through western blot. **** P<0.0001, ** P<0.01, * P<0.05.

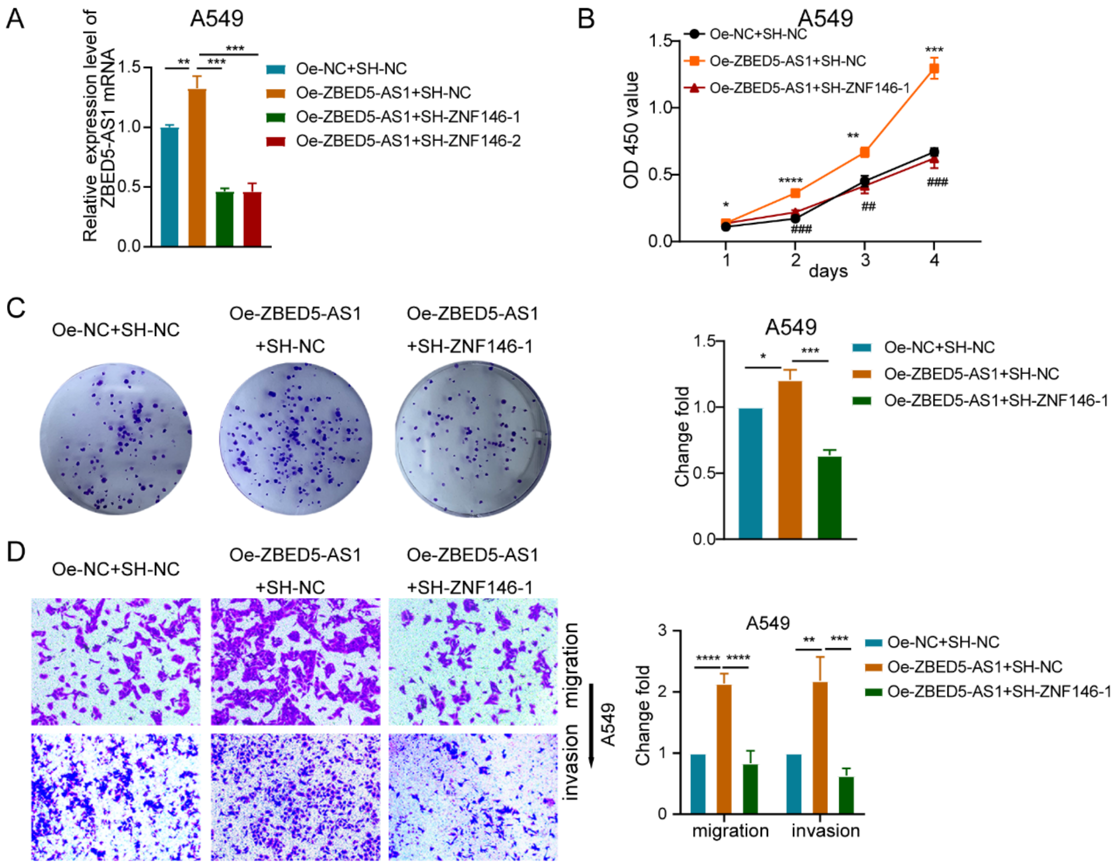

Figure S5. ZBED5-AS1 promotes the proliferation and invasion of LUAD cells by ZNF146 in A549 cells. (**A**) ZNF146 expression after co-transfection of Oe- ZBED5-AS1 with SH- ZNF146 in A549 cells by RT-qPCR. (**B**) CKK-8 assay analysis of LUAD cells proliferation after co-transfected with Oe- ZBED5-AS1 and SH-ZNF146 in A549 cells. (**C**) The colony formation assays analysis of LUAD cells proliferation after co-transfected with Oe- ZBED5-AS1 and SH-ZNF146 in A549 cells. (**D**) Migration assay and Invasion assay analysis of LUAD cells migration effect of A549 cells after co-transfected with Oe- ZBED5-AS1 and SH-ZNF146 in H1299 cells. **** P<0.0001, *** P<0.001, ** P<0.01, * P<0.05.
