# Supplementary material for: Long Noncoding RNA ZBED5-AS1 Facilitates Tumor Progression and Metastasis in Lung Adenocarcinoma via ZNF146/ATR/Chk1 Axis
Source: Int J Mol Sci. 2023 Sep 10;24(18):13925. doi: 10.3390/ijms241813925 (PMC10530271; doi:10.3390/ijms241813925)
Supplement: Supplementary file 1 [file ijms-24-13925-s001.zip › Supplementary Table S1.pdf]

Supplementary Table S1 Primer sequence of genes

**Primer（F）**	**Sequence(5'to3')**	**Primer (R)**	**Sequence(5'to3')**
ZBED5-AS1-F	CTCAAACTTCACTAGCCCCGT	ZBED5-AS1-R	GAATGGGATGGGAGGACACA
ZNF146-F	TTTGGTCTTTGTCCGCGGGT	ZNF146-R	TCTTCTCAGGCTTCTTCCGC
ATR-F	TGTCTGTACTCTTCACGGCATGTT	ATR-R	AAGAGGTCCACATGTCCGTGTT
Chk1-F	GGTGAATATAGTGCTGCTATGTTGACA	Chk1-R	TTGGATAAACAGGGAAGTGAACAC
β-actin-F	CCTGGCACCCAGCACAAT	β-actin-R	GCTGATCCACATCTGCTGGAA
U6-F	AGAGAAGATCGGCATGGTTACTG	U6-R	ACCTGAGCGCATTGTCCGAGG
GAPDH-F	GACGCGTGGGAACATGAGACA	GAPDH-R	ATGACCTGGACGGTCGAGCGC
